# NKG2D and Its Ligands: “One for All, All for One”

**DOI:** 10.3389/fimmu.2018.00476

**Published:** 2018-03-12

**Authors:** Alessandra Zingoni, Rosa Molfetta, Cinzia Fionda, Alessandra Soriani, Rossella Paolini, Marco Cippitelli, Cristina Cerboni, Angela Santoni

**Affiliations:** ^1^Department of Molecular Medicine, Sapienza University of Rome, Laboratory Affiliated to Istituto Pasteur Italia-Fondazione Cenci Bolognetti, Rome, Italy

**Keywords:** NKG2D ligands, NKG2D receptor, NK cells, virus infection, cancer

## Abstract

The activating receptor NKG2D is peculiar in its capability to bind to numerous and highly diversified MHC class I-like self-molecules. These ligands are poorly expressed on normal cells but can be induced on damaged, transformed or infected cells, with the final NKG2D ligand expression resulting from multiple levels of regulation. Although redundant molecular mechanisms can converge in the regulation of all NKG2D ligands, different stimuli can induce specific cellular responses, leading to the expression of one or few ligands. A large body of evidence demonstrates that NK cell activation can be triggered by different NKG2D ligands, often expressed on the same cell, suggesting a functional redundancy of these molecules. However, since a number of evasion mechanisms can reduce membrane expression of these molecules both on virus-infected and tumor cells, the co-expression of different ligands and/or the presence of allelic forms of the same ligand guarantee NKG2D activation in various stressful conditions and cell contexts. Noteworthy, NKG2D ligands can differ in their ability to down-modulate NKG2D membrane expression in human NK cells supporting the idea that NKG2D transduces different signals upon binding various ligands. Moreover, whether proteolytically shed and exosome-associated soluble NKG2D ligands share with their membrane-bound counterparts the same ability to induce NKG2D-mediated signaling is still a matter of debate. Here, we will review recent studies on the NKG2D/NKG2D ligand biology to summarize and discuss the redundancy and/or diversity in ligand expression, regulation, and receptor specificity.

## Introduction

NK cells and T cells respond to pathogens and tumors by the integration of signals deriving from numerous cell surface receptors that can initiate, enhance or suppress lymphocyte effector functions. While the antigen-specific T cell receptor—generated by somatic genetic recombination—dictates T cell recognition and activation, NK cells use a vast repertoire of germ-line encoded receptors. Many of them are also expressed by T cells, with NKG2D being one of the best characterized receptors shared by both cell types (1).

NKG2D is a C-type lectin-like receptor expressed on NK cells, γδ T cells, CD8^+^ T cells, and some autoreactive or immunosuppressive CD4^+^ T cells and represents a major recognition receptor for the detection and elimination of damaged, transformed, and pathogen-infected cells. Its ligands belong to the H60 (a–c), RAE (α–ε), and MULT1 families in mice, and to the MIC (MICA and MICB) and ULBP (ULBP1–ULBP6) families in humans, where their repertoire is more complex than in other species (1, 2). In fact, MIC molecules are encoded by the most highly polymorphic human genes after the classical HLA molecules, while murine ligands have a low allelic diversity (2).

But which is the reason and biological significance of having more than 100 different alleles for MICA, 40 for MICB, and less than 20 for the ULBPs, or, in other words, why do we have such a diversity of NKG2D ligand genes and alleles? The answer lies in the million years of co-evolution between host and pathogens. “Cat and mouse” evolution of the host immune system and pathogen immune evasion mechanisms are a dominant view of immunogenetics, and among the different classes of pathogens, there is ample evidence that viruses, in particular herpesviruses, could be the major driving force for the evolution of NKG2D ligand diversity. Moreover, gene duplications and further mutations within the alleles result in such a ligand variety that there is not a single viral protein or RNA described so far able to target all of them (2, 3). Thus, possessing a high number of NKG2D ligands together with genetic polymorphisms is clearly advantageous to the host, allowing it to counteract viral immune evasion strategies. In fact, in the paradigmatic example of cytomegalovirus, although viral immune evasion genes are strongly diversified, they are not entirely successful, since NK cells are functionally active, with NKG2D playing a role in the elimination of infected cells (4–8). On the other hand, there is no convincing evidence to date that non-viral infectious pathogens are a significant drive for the evolution for NKG2D ligand diversity, and for the “cat and mouse” competition, thus further supporting a role for the viral-mediated selective pressure.

Although the origin and evolution of NKG2D ligand variety dates back to host–pathogen competition, infections are far to be the only examples in which NKG2D ligands are induced. In fact, they can be expressed at different levels on some normal cells (9–11), but more typically they are upregulated on tumor cells (12). The pathways underlying the regulation of their expression are generally activated by different forms of stress and cellular abnormalities, often associated with tumor transformation and progression, and they can act at different levels: regulation of transcription and protein synthesis, posttranslational modifications, and release of ligands in the microenvironment have been all described as important mechanisms controlling NKG2D ligand expression (13). Thus, primary tumors frequently express NKG2D ligands but, as in the case of viral infections, several mechanisms have been identified that elude the detection and elimination of tumor cells by the immune system, suggesting an NKG2D-mediated immune editing of the tumor (14). As with virus-infected cells, alerting the immune system will be then the final result of a balance between expression of ligands and tumor immunoevasion strategies (15).

Yet, although we have many information today on both the NKG2D receptor and its numerous ligands, some key questions still await a full comprehension and exhaustive answer. Are the ligands regulated in different manner depending on the cell type, stimulus, microenvironment, etc? And if so, are there general rules for which the different ligands are induced in various cell types by different stimuli? Do different ligands and/or alleles bind to NKG2D with different affinities? Are the ligands redundant in their functions? Or are they specific?

In this review, we will try to discuss these aspects, illustrating the diversity of ligand expression, regulation, and receptor specificity, in the context of viral infections and tumor transformation.

## NKG2D Ligand Regulation: Redundancy and Specificity

NKG2D ligand expression at the cell surface results from multiple levels of regulation, and as a consequence, it is often due to the contribution of distinct pathways acting collectively. We will discuss the specialization of some regulatory processes intrinsic to one or few ligands, as well as the redundancy of other molecular mechanisms able to simultaneously control the expression of several NKG2D ligands in normal conditions and in response to different stimuli. Moreover, most of the studies reported that modulation of NKG2D ligand expression on “target” cells affected NK cell recognition and killing (Table 1).

**Table 1:** Mechanisms regulating NKG2D ligand expression in steady state conditions and in response to stimuli.

Regulatory level	Condition/stimuli	Pathway/molecule	Ligand modulation	Cell type	NK cell functions	Reference
**TRANSCRIPTIONAL**						
	Ionizing radiation, cisplatin, and 5-FU	DDR	↑ MICA, ULBP1–3 ↑ RAE-1, MULT1	HFF; C1, C2 cell lines	↑ Cytotoxicity	(16)

	Doxorubicin and melphalan	DDR/E2F1	↑ MICA	Multiple myeloma	↑ Cytotoxicity ↑ IFNγ	(17, 18)

	Activation/proliferation	NF-κB NF-κB E2F1	↑ MICA ↑ MICA ↑ RAE-1ε	T cells T cells Primary fibroblasts, embrionic brain cells	n.d. ↑ Cytotoxicity ↑ Cytotoxicity	(9) (19) (20)

	Ara-C	STING/TBK/IRF3	↑ RAE-1	B cell lymphoma	↑ Cytotoxicity	(21)

	RITA Vincristine	p53 p53 p53	↑ ULBP1, ULBP2 ↑ ULBP2 ↑ ULBP1	Cancer cell lines Cancer cell lines Multiple myeloma	↑ Cytotoxicity ↑ IFNγ ↑ Cytotoxicity ↑ Cytotoxicity	(22) (23) (24)

	Heat shock response	HSF1	↑ MICA, MICB	Cancer cell lines Multiple myeloma	n.d. ↑ Cytotoxicity	(25) (26)

	ER-induced stress	CHOP	↑ ULBP1 ↑ MULT1	Intestinal epithelial cells	↑ Cytotoxicity	(27)

	Steady state	STAT3	↑ MICA	Colonrectal cancer Multiple myeloma	↑ Cytotoxicity ↑ IFNγ ↑ Cytotoxicity	(28) (29)

	Steady state	IKZE1/3, IRF4	↑ MICA	Multiple myeloma	↑ Cytotoxicity	(30, 31)
**RNA SPLICING**						
	Steady state	RBM4	↓ ULBP1	HAP1 cell line	n.d.	(32)
**POSTTRANSCRIPTIONAL**						
	Steady state	AUF1	↓ ULBP2, MICB	Epithelial cells	n.d.	(33)

	Steady state	miR34a, c miR-519a-3p miR-93 miR-20a	↓ ULBP2 ↓ MICA, ULBP2 ↓ MICA, MICB, ULBP3 ↓ MICA, MICB, ULBP2	Melanoma cell lines Mammary epithelial cell line Glioma cell lines Cancer cell lines	↓ Cytotoxicity ↓ Cytotoxicity ↓ Cytotoxicity ↓ Cytotoxicity	(34) (35) (36) (37, 38)

	IFNγ	miR-520b	↓ MICA	Cancer cell lines	n.d.	(39)

	HCMV, KSHV, and EBV	miR-UL112; miRK12-7; and miR-BART2-5	↓ MICB	Infected HFF cells; cancer cell lines	↓ Cytotoxicity	(40, 41)

	JCV	miR-J1-3p	↓ ULBP3	Infected cancer cell lines	↓ Cytotoxicity	(42)
**Posttranslational**						
	Steady state	Ubiquitination	↓ MULT1	Cancer cell lines	n.d.	(43)

	KSHV	K5 ubiquitin ligase	↓ MICA	Cancer cell lines	↓ Cytotoxicity	(44)

	Histamine	Ubiquitination	↓ MICA	Monocytic leukemia	↓ Cytotoxicity	(45)

	Steady state	ADAMs and MMPs (protease-mediated shedding)	↓ MICA, MICB, ULBP2	Cancer cell lines	↓ Cytotoxicity n.d.	(46–48) (49–51)

*↓ Decrease; ↑ increase; HFF, human foreskin fibroblasts; HCMV, human cytomegalovirus; EBV, Epstein–Barr virus; KSHV, Kaposi’s sarcoma-associated herpes virus; JCV, human polyoma virus JC; ADAM, a disintegrin and metalloprotease; MMP, matrix metalloprotease; n.d., not done; CHOP, C/EBP homology protein*.

*Ref. (48) provides an ample overview on protease-mediated shedding of NKG2D ligands*.

## Transcriptional Regulation

NKG2D ligands can be regulated at transcriptional level by a plethora of molecular pathways; both the multiplicity of transcription factors (TFs) and the diversity in the regulatory sequences in NKG2D ligand gene promoters can significantly contribute to generate the extensive heterogeneous expression of these proteins in different cell types. Indeed, distinct TFs are able to regulate the transcription of a number of NKG2D ligands in different systems; on the other hand, some cell lineage specific transcriptional regulators of selected ligands have also been described in cancer cells. Finally, epigenetic mechanisms have a robust impact on the transcriptional regulation of diverse NKG2D ligands.

NKG2D ligand-inducing cell stresses, including proliferative signals, malignant transformation, infection, or oxidative stress, share the ability to activate a DNA damage response involved in maintaining the integrity of the genome (52, 53). In this context, the sensor kinases ATM and ATR can trigger a signaling cascade in which different downstream checkpoint kinases, such as Chk1 and Chk2, are activated together with the key tumor suppressor p53 (54). Interestingly, although the activity of these kinases is needed for the induction of MIC, ULBPs or Raet1 genes (16, 17), p53 can differently regulate several NKG2D ligands, e.g., ULBP1–2, with no evident effects on the expression of MICA/B (22–24). On the other hand, the same pathway connected with proliferative signals (19, 20) or triggered by oxidative stressors (18) can enhance the activity of either NF-κB or E2F1, thus inducing the transcription of MICA/B in humans, or RAE-1 in the mouse. In addition, genomic damage or cytosolic DNA can lead to the activation of the DNA sensor pathway regulated by STING/TBK1 and IRF3, identified as regulators of RAE-1 ligands (21). These observations suggest that triggering of DDR (16), together with the induction of a senescence program (17, 18, 55) and/or the involvement of cytosolic DNA/RNA sensors (21), represents a major signal of activation/alarm for NKG2D-expressing cells (e.g., NK cells), likely establishing a primary checkpoint for aberrant cell proliferation or infection. Of note, the expression and/or function of effector proteins of these pathways can be often altered/defective in cancer cells, suggesting that their contribution in the regulation of one or more specific ligands guarantees the expression of these molecules in different types of tumors or at different disease stages.

Mechanisms of chromatin remodeling are also widely implicated in the transcriptional regulation of almost all NKG2D ligands. Both hypomethylating agents and histone deacetylase inhibitors have been shown to upregulate MICA/B and ULBPs surface levels in different tumors and infected cells (56–61), thus indicating that transcriptional silencing of these genes is largely dependent on events of DNA methylation and deacetylation. Accordingly, epigenetic dysregulation of NKG2D ligand promoters is an important immune evasion mechanism helping cancer or infected cells to acquire resistance to NK cell surveillance.

An example of a molecular pathway selectively targeting MICA and MICB molecules is the heat-shock response that regulates their expression in different models. In this regard, ChIP experiments indicated that the MICA/B promoters are occupied by the HSF1 in heat-shocked cells, or in cells where the basal repression of this factor by the chaperone HSP90 is abrogated by specific small molecule inhibitors or proteotoxic stress (25, 26). Although promoter sequence analyses indicate that potential canonical heat-shock elements also exist in some of the ULBP genes (62, 63), no evidence regarding heat shock-induced ULBP expression has been reported to date.

In a different context, accumulation of improperly folded proteins or alterated UPR, as shown in dysregulated intestinal epithelial cells and human cell lines, can induce the selective expression of diverse ULBPs *via* C/EBP homology protein-mediated transactivation, and increase NKG2D-mediated epithelial cytolysis (27). In addition, a critical role for the TF ATF4 has been also described for UPR-induced upregulation of ULBP1 in human cell lines (32).

Different mechanisms of transcriptional repression have been described only for MICA; they are caused by cell type-specific proteins, the expression and/or function of which is often critical for the survival and proliferation of cancer cells. A cogent example is the TF STAT3 shown to directly interact with MICA promoter and repress its transcription in colon cancer cells (28); the same mechanism was found to occur also in multiple myeloma cells where the serine–threonine kinase GSK3 was identified as an important upstream regulator of STAT3 contributing to the inhibition of MICA expression (29). Furthermore, three TFs highly expressed in multiple myeloma and pivotal regulators of malignancy-specific gene expression, the Ikaros family zinc finger protein-1 and -3 (IKZF1 and IKZF3) and IRF4, are potent repressors of MICA expression in this hematological cancer (30, 31).

Connected with these mechanisms able to negatively regulate the expression of NKG2D ligands at transcriptional level is the polymorphism of their promoters. It is well known that promoters of MICA and MICB are polymorphic (12 MICA/B promoter haplotypes) with some polymorphisms associated with reduced expression (64, 65) or increased susceptibility to specific diseases (66). Accordingly, binding sites for TFs in MICA/B promoters have been demonstrated to be interrupted by polymorphisms within these regions, resulting in allele-specific regulation (65). These findings indicate that individuals with the same alleles might show variation in the expression of MICA and MICB because of polymorphism in their promoters. Altogether, these modifications may lead to selective transcriptional regulation of distinct NKG2D ligands.

In summary, despite most of the transcriptional regulatory mechanisms are common to distinct ligands, assuring the recognition and elimination of stressed cells, a number of studies indicate a certain level of specificity in the type of “stressor” that allows the expression of a particular ligand. Heat-shock response acts essentially on MICA/B but not on ULBPs (25); ligand induction in proliferating cells is characteristic of some NKG2D ligands like RAE-1 but not MULT1 or H60 (20) and in proliferating T cells MIC molecules are induced with a faster kinetic when compared with ULBPs (19, 67); ER-induced stress results in selective upregulation of ULBP1 in humans and MULT1 in mice (27). Thus, different stimuli induce specific cellular responses leading to the expression of one or few ligands, originating a sophisticated mechanism to alert the immune response.

## RNA Splicing

Regulation of RNA splicing represents another mechanism to control NKG2D ligand expression. In particular, Gowen et al. have shown that the RNA-binding protein (RBP) RBM4 supports ULBP1 expression by suppressing a novel alternatively spliced isoform of ULBP1 mRNA and appears to be specific for the differential splicing of ULBP1 but not of other NKG2D ligands (32). Although alternative splicing isoforms have been described for MICA (68), ULBP4 (69) and ULBP5 (70), the molecular mechanisms involved in their regulation is still unknown.

## Posttranscriptional Regulation

Stabilization of NKG2D ligand mRNA is considered an important mode to strictly control ligand expression mainly under normal conditions. In this context, a new pathway by which NKG2D ligand mRNAs (i.e., MICA, MICB, and ULBP2) are constitutively targeted by AUF1 proteins that mediate RNA degradation has been identified (71). In response to EGFR activation, either by its ligand or by some type of stress, AUF1 molecules are excluded from the nucleus allowing NKG2D ligand mRNA to be stabilized. In addition, the oncogenic RBP IMP3, which selectively binds to ULBP2 but not ULBP1 and ULBP3 mRNA, leads to ULBP2 transcript destabilization and reduced ULBP2 surface expression in several human cell lines (33). Similarly, Nachmani et al. identified other RBPs able to bind to MICB RNA and regulate its expression (72).

A number of studies have shown that distinct NKG2D ligands are regulated by microRNAs (miRNAs), which are short, non-coding RNAs that exert their regulation of gene expression posttranscriptionally by targeting 3′-untranslated region (3′UTR) of the target mRNAs and leading to degradation or translation inhibition (73). Different sites for cellular miRNAs within the 3′UTRs and/or the 5′UTR of MICA, MICB, and ULBP1 have been identified (39, 74–77). Interestingly, several viruses use miRNAs to hinder NKG2D ligand expression and evade the NKG2D-dependent immunosurveillance. Indeed, Stern-Ginossar and colleagues identified a group of endogenous cellular miRNAs regulating MICB and MICA expression by targeting a specific site also used by the human cytomegalovirus (HCMV) miRNA miR-UL112 (40, 74). Despite MICA and MICB display almost identical putative binding sequence for miR-UL112, the cooperation between miR-UL112 and cellular miRNA was reported to suppress only MICB expression during HCMV infection, suggesting that additional factors are involved in determining a functional binding site (40). Similarly, results of functional approaches and basic bioinformatic tools demonstrated that other herpesvirus miRNAs (i.e., Kaposi’s sarcoma-associated herpesvirus, KSHV, and Epstein–Barr virus) downregulate preferentially MICB but not MICA expression (41). The human polyoma viruses BKV and JCV use an identical miRNA to evade NK cell control by downregulating the stress-induced ligand ULBP3 (42).

Moreover, miRNA-mediated NKG2D ligand regulation occurs also in cancer cells. Indeed, the tumor-suppressive miR34a and miR34c strongly downregulated ULBP2 in human melanoma (34). The downregulation of ULBP2 and MICA expression by miR-519a-3p has been implicated in the inhibition of NK cell-mediated cytotoxicity of breast cancer cells (35), whereas miR-93 mimics decreased cell surface expression of MICA, MICB, and ULBP3 by translational repression, thus contributing to the immune evasion of glioma cells (36). Recently, miR-17-92 cluster was also reported to downregulate MICA/B protein expression in ovarian tumors (37), and breast cancers (38). In the latter, the authors provided the evidence that miR-17-92 members affected ULBP2 expression by inhibiting the MAPK/ERK signaling pathway (38). Finally, also some cytokines regulate NKG2D ligand expression by miRNAs. In particular, IFN-γ increased expression of miR-520b able to inhibit MICA transcript levels in different types of cancer cell lines (39).

All together these studies highlight the fact that cellular miRNAs and RBPs represent an important way to keep a low NKG2D ligand expression in steady state conditions, and they emerge as a general mechanism to regulate both ULBPs and MIC molecules.

## Posttranslational Regulation

The surface expression levels of a determined NKG2D ligand can be finely controlled by mechanisms implicated in the regulation of its release as soluble form by various processes including protease-mediated cleavage, exosome secretion, and alternative splicing. The choice of one of these processes is mainly dependent on the ligand type as well as its allelic variant.

In general, both MIC and ULBP molecules are cleaved by proteases belonging to two distinct families, the matrix metalloproteinases (MMPs) and a disintegrin and metalloproteases (ADAMs) (46–51) that undergo modulation of their activity and expression (78–81). Different susceptibility to the protease-mediated cleavage has been described for several NKG2D ligands. As such, ULBP1, ULBP2, and ULBP3 are released from cells with different kinetics and by distinct mechanisms, being ULBP1 and ULBP3 more resistant to cleavage and preferentially secreted into exosome-like vesicles (82). Similarly, the short-allelic variant MICA*008, the prototype of a group of MICA alleles named MICA*A5.1, is resistant to proteolytic cleavage and mostly released from cells in association with exosomes (83). Also the MICA-129 dimorphism, causing a valine to methionine exchange at position 129, has been described to affect MICA shedding, but the mechanism behind is largely unknown (84). Thus, the existence of NKG2D ligands and/or allelic variants with distinct sensitivity to proteases might have relevant functional consequences. In this regard, genotoxic agents have been reported to selectively stimulate the shedding of MICB or of the allelic variant MICA*019 in a ADAM10-dependent manner, whereas the release of the short MICA*008 allele was not perturbed. Therefore, during the course of chemotherapy, MICA*008 appears to be more stable on the tumor cell surface thus favoring the recognition and killing by NK cells (85). In another study, expression of the metalloprotease tissue inhibitor of metalloproteinase 3 (TIMP3), induced by specific miRNAs in HMCV-infected cells, resulted in an enhanced activity of ADAM17 and MMP14 and increased MICA shedding (86). Moreover, an increased protease-mediated shedding of MICA, MICB, and ULBP2 was described in HIV-infected CD4^+^ T cells (87).

On the other side, the soluble form of the high-affinity mouse NKG2D ligand, MULT1, promotes NK cell activation and tumor rejection (88). Indeed, in an *in vivo* mouse model, Deng and coworkers reported that in the presence of this soluble ligand, NK cells were not desensitized because soluble MULT1 prevented the chronic interactions between NKG2D and its ligands on cells of the tumor microenvironment (88). However, these effects appear to be restricted to the mouse and could depend on the capacity of MULT1 to bind to NKG2D with an elevated affinity respect to other mouse NKG2D ligands.

In regard to the exosome secretion, both MIC and ULBP ligand family members can be released by this class of nanovesicles (89–91), and some of them such as ULBP3 or ULBP1 (82) or the allelic variant MICA*008 (83) are secreted exclusively by exosomes. Interestingly, increased exosome secretion has been observed in response to different types of stress (90, 92, 93); however, unlike the protease-mediated shedding, it is still unclear whether the release of NKG2D ligands *via* exosomes could result in the reduction of their surface expression.

In addition to shedding and exosomal secretion, alternative splicing represents another mode to generate soluble forms of some ligands as demonstrated for ULBP4 and ULBP5 (69, 70).

Several reports have provided also evidence that other posttranslational mechanisms concur to regulate NKG2D ligand expression at protein level, including protein turnover and ubiquitination. For instance, stability of ULBP1 and MICB at the plasma membrane is lower than for other ligands, and in part occurs because of a rapid internalization (94, 95). The intracellular sequestration of immature forms of MICA in the endoplasmic reticulum was observed in melanoma cancer cells and proposed as an immune escape strategy (96).

Reduction of MICA expression by ubiquitination has been described in different models (44, 45). It is used as evasion strategy by KSHV because it encodes the K5 ubiquitin ligase that ubiquitinates MICA cytoplasmic tail, thus causing a profound downregulation of this ligand on the surface of infected cells (44). Of note, the truncated allelic variant MICA*008, lacking lysine residues in its cytoplasmic tail, was resistant to KSHV-induced downregulation suggesting a selective advantage for individuals carrying such allele. Moreover, it has been shown that the murine ligand MULT1 is ubiquitinated and degraded in normal cells, and this process is reduced in response to heat shock or ultraviolet irradiation (43). Thus, targeting the ubiquitination machinery in cancer or virus-infected cells might increase their susceptibility to NK cell-mediated killing.

In conclusion, structural characteristics inherent to a specific ligand/allelic variant affecting the different susceptibility to the protease-mediated cleavage, the ubiquitination-mediated degradation and its stability on the cell surface, represent all fundamental elements to successfully complete the long route that finally allows the ligand to be expressed on the cell surface.

## Distinct Ligands or Allelic Variants Differ in Their Ability to Modulate NKG2D-Mediated Signaling

Given that NKG2D ligands are characterized by variable domain structure, distinct mode of membrane anchor and diverse affinity for their receptor, it is likely that they are not equally able to evoke activating signals. This paragraph summarizes recent findings that support the capability of NKG2D ligands to differently regulate NKG2D signaling events in both NK and T cells.

Engagement of human NKG2D elicits cytolytic responses overcoming inhibitory signals on NK cells and enhancing TCR-dependent activation in CD8^+^ T cells, Vγ2Vδ2, and gut intraepithelial Vγ1δ1 T cells (97–100). In CD8^+^ T cells, both co-stimulatory and T cell receptor independent functions have been described (101–104), whereas Vγ9Vδ2 T cells can be directly activated by NKG2D in the absence of TCR-dependent antigen recognition (105). Of note, NKG2D engagement alone can elicit effector functions only in NK cells preactivated by cytokines including IL-15 (106) and IL-2 (107), whereas the synergistic engagement of at least another activating receptor is required on freshly isolated NK cells (108). Thus, NKG2D can provide either a co-stimulatory signal or a direct activating signal depending on the cell context and/or the initial influence of cytokine environment.

In humans, NKG2D homodimer forms a hexameric complex with two homodimers of the transmembrane adaptor DNAX-activating protein 10 (DAP10), which is involved in intracellular signal propagation (109). Indeed, the cytoplasmic tail of DAP10 contains a tyrosine based signaling motif (YINM), which is tyrosine phosphorylated by Src-family kinases upon antibody-mediated NKG2D engagement (110). DAP10 phosphorylation promotes the recruitment of the p85 regulatory subunit of PI3K and of the Grb2/Vav1 complex, that, in turn, is required for the phosphorylation of Src homology 2 domain-containing leukocyte protein of 76 kD (SLP-76) and of phospholipase C gamma (PLCγ2) (109–111).

Even though the interaction with target cells exposing one or more NKG2D ligands triggers a functional response, the relative contribution of distinct ligands to specific signaling pathways remains elusive.

Of note, persistent stimulation with membrane-bound or soluble NKG2D ligands down-modulates receptor expression and ultimately impairs NKG2D-dependent functions on both NK and CD8^+^ T cells (46, 91, 112–115). This functional impairment is achieved by a rapid NKG2D internalization from plasma membrane and sorting along the endocytic compartments till lysosomes, where internalized receptor complexes are degraded (113, 116–118).

In addition to reduce surface receptor expression, NKG2D endocytosis also plays an indispensable role in NKG2D-mediated signaling. Indeed, recent findings demonstrate that receptor endocytosis is required for cytotoxic granule secretion and IFNγ production. In particular, the activation of extracellular signal-regulated kinases 1 and 2 (ERK1 and ERK2) was found to occur in signaling-competent endocytic compartments where the internalized complexes are also transported, demonstrating that NKG2D continues to signal before reaching lysosomes for degradation (119). Whether distinct ligands differ in their ability to promote signaling from endosomes is currently unknown.

**Figure 1:**
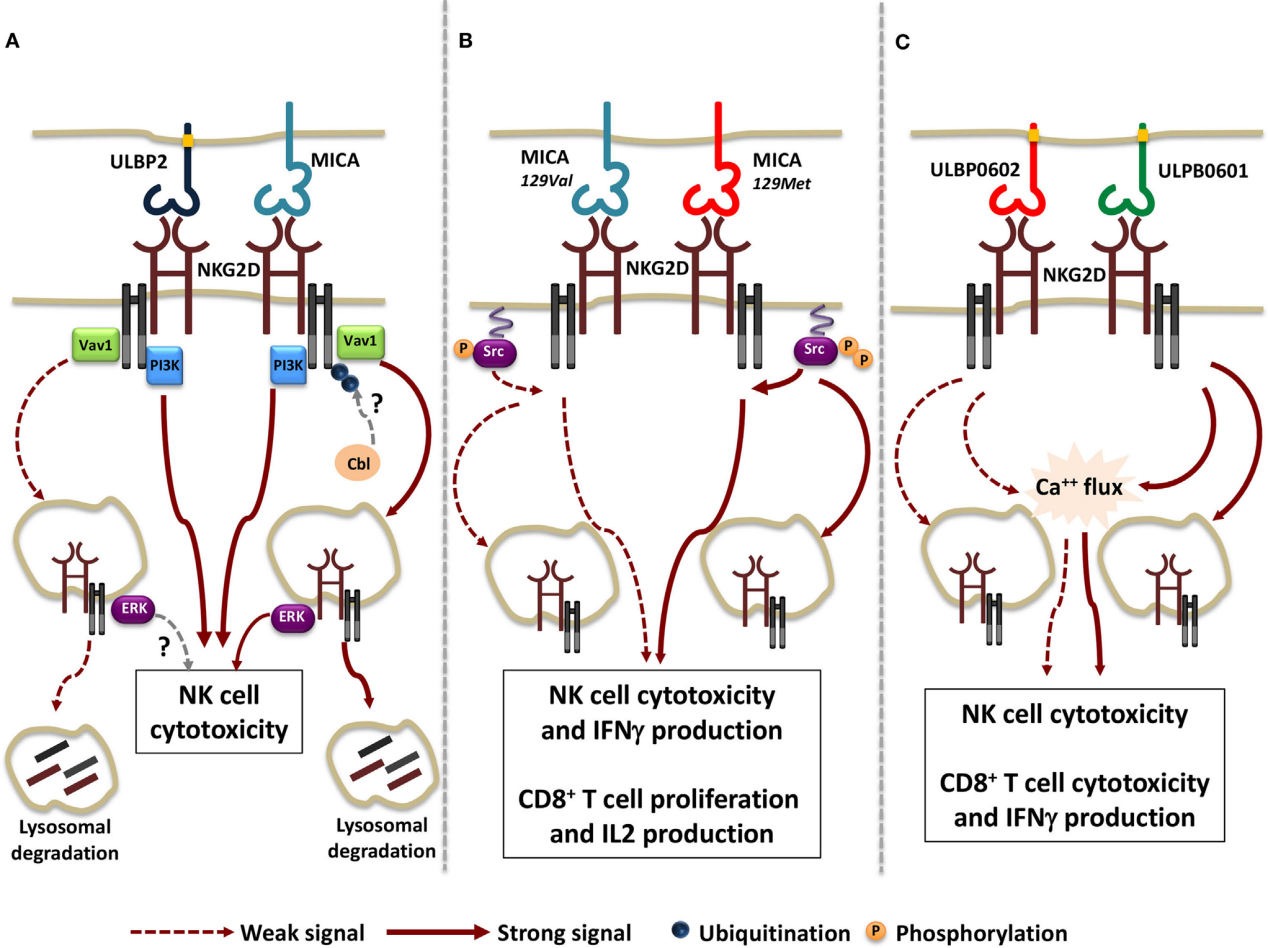
Functional consequences of the interaction of NKG2D with different ligand/allelic variants. **(A)** Transmembrane MICA and GPI-linked ULBP2 ligands result equally able to trigger Vav1 and PI3K activation and to induce NK cell cytotoxic function. However, a stronger receptor internalization and lysosomal degradation due to the activation of the ubiquitin pathway was observed upon MICA engagement. Whether Cbl is the ubiquitin ligase regulating NKG2D/DNAX-activating protein 10 ubiquitination and whether ULBP2 ligand is able to activate NKG2D-mediated signals from endosomal compartment is not clear (dashed arrows). **(B)** MICA-129Met, which binds to NKG2D with higher avidity compared with MICA-129Val allele, induces stronger Src phosphorylation, thus triggering both NK cell and CD8^+^ T cell effector functions with higher efficiency. Concomitantly, a higher extent of NKG2D down-modulation is also induced upon MICA-129Met allele engagement. **(C)** The rigid and stable binding to NKG2D of the high-affinity ULBP0602 variant impairs its ability to induce Ca^++^ flux and effector functions in NK cell and CD8^+^ T cells as well as NKG2D down-modulation. As consequence, ULBP0602 engagement results less efficient compared with the low affinity variant ULBP0601.

Regarding the extent of receptor internalization and the rate of NKG2D lysosomal degradation, the nature of the ligand appears to play a pivotal role, as demonstrated by comparing the ability of membrane-bound MICA and ULBP2 to regulate NKG2D expression (118). Indeed, MICA promotes a more rapid NKG2D down-modulation if compared with ULBP2, leading to a stronger lysosomal degradation (Figure 1A). Although MICA and ULBP2 resulted equally able to elicit NKG2D-mediated NK cell cytotoxic function, the ability to further perform cytotoxicity resulted dramatically impaired only upon MICA-induced NKG2D down-modulation.

All together these results suggest that distinct ligands have the potential to activate selective signaling pathways resulting in different routes of receptor endocytosis. To this regard, we have reported that phosphorylation of the ubiquitin ligase c-Cbl, a negative regulator of NKG2D signaling (120), and the activation of the ubiquitin pathway is indispensable for MICA- but not ULBP2-induced NKG2D internalization and degradation (Figure 1A) (118). This selective behavior can be either attributable to MICA and ULBP2 distinct mode of membrane anchor (transmembrane and GPI-linked, respectively) and/or to differences in their affinity/avidity for NKG2D. To this regard, recent evidences demonstrate that allelic variants of the same ligand that differ in their avidity for NKG2D can also vary in their ability to tune the threshold of NKG2D signaling (121, 122).

Regarding MICA, the dimorphism in the position 129 strongly affects ligand ability to promote NK cell effector functions and to co-stimulate CD8^+^ T cell activation (121). MICA-129Met allele binds to NKG2D with higher avidity than MICA-129Val variant (123) and appears to be more efficient in the induction of proximal signaling events such as Src phosphorylation and in triggering NK cell degranulation and IFNγ release (121). In CD8^+^ T cells, MICA-129Met co-stimulates IL-2 production and proliferation with a more rapid kinetic than the MICA-129Val variant. On the other hand, NKG2D engagement by the MICA-129Met isoform results in a stronger receptor down-modulation in both NK and T cells leading to a severe impairment of NKG2D-mediated functions and avoiding excessive cell activity (Figure 1B).

By comparing the allelic variants of ULBP6, the most polymorphic ULPB ligand (124, 125), the amino acid substitution (from Arg to Leu) in position 106 reported in the ULBP0602 variant was found to be responsible for the great enhancement in affinity and stability of NKG2D interaction compared with the ULBP0601 allelic variant (122). Unexpectedly, the higher affinity variant resulted less able to elicit both NKG2D down-modulation and functional responses in NK cells as well as in CD8^+^ T cells and γδT cells (Figure 1C). To explain these findings, the authors speculated that a rigid and stable interaction with the higher affinity ligand ULBP0602 limits the ability of cytotoxic lymphocytes to serially kill their targets.

Collectively, these results outline a hierarchy of cellular responses to different allelic variants of NKG2D ligands, suggesting that they elicit heterogeneous functional outcomes (Figures 1B,C). In addition, they support the notion that the strength of NKG2D-mediated signaling positively correlates with the rapidity and degree of receptor down-modulation. The interconnection between signaling and endocytosis guarantees a rapid and tight regulation of NKG2D activation preventing strong intracellular signals that could drive autoimmune responses.

A further level of complexity is given by the potential ability of soluble NKG2D ligands to modulate NKG2D signal propagation by regulating receptor surface expression. Several lines of evidence have demonstrated that the presence of soluble ligands in the sera of neoplastic patients correlates with a reduced NKG2D surface expression (113, 114, 126), suggesting that soluble NKG2D ligands share with their respective membrane-bound forms the ability to regulate receptor expression. However, a direct comparison between ligands shed after proteolytic cleavage and ligands released in exosomes, demonstrate a higher ability of the latter to induce receptor down-modulation. Regarding MICA, the GPI-linked allele MICA*008 that is released in association with exosome membranes, is a more potent NKG2D down-modulator compared with metalloproteinase-shed MICA variants (83). Accordingly, exosome-released ULBP3 molecules reduce NKG2D surface expression and compromise NKG2D-mediated NK cell cytotoxic function with higher efficiency than the metalloproteinase-shed ULBP2 ligands (82). These results may be explained by the presence of ligands on exosomal membranes that can multimerize and bind to NKG2D with higher avidity than the soluble counterpart. Whether the ability of exosomal multimeric ligands to efficiently down-modulate receptor expression reflects their ability to induce intracellular signals and elicit selective functional responses is currently unknown.

## Conclusion

Diversified modalities of NKG2D ligand regulation can be applied to all NKG2D ligands while others are specific just for one or a few of them. In steady state conditions, NKG2D ligand expression is tightly repressed to maintain immune homeostasis. In response to external “danger” signals (i.e., stress and pathogens) or during neoplastic transformation, increased transcriptional activity of NKG2D ligand genes together with a perturbation of their regulatory mechanisms at mRNA and protein levels leads to the ultimate ligand cell surface expression. The sum of critical factors (the type of stressor, the structural characteristics of the ligand/allelic variant and the cellular context) determines the expression of one or more but not all ligands on the cell surface. Thus, despite some common regulatory mechanisms, NKG2D ligands are not equal or redundant in terms of final outcome, and the “specificity” of these cellular responses triggered by a multiplicity of ligands assures a sophisticated mechanism to alert the immune response. The “one for all” aspect is another compelling and still unsolved issue of the NKG2D-dependent immunosurveillance, where the large variety of ligands appears functionally non-redundant, even though they all engage the same receptor. In this regard, the expression of a certain ligand or a particular allelic variant is essential to drive a proper immune response. To date, many questions still remain open, and it is unclear if a hierarchy exists and if one of them can dominate in the triggering ability when distinct ligands have reached the cell surface; alternatively it is plausible that the ligands may act “all for one,” contributing in concert to the NKG2D-mediated functional response. Thus, a detailed characterization of the cell biology of single NKG2D ligand will be indispensable to warrant targeted modulation of this system in the course of a viral infection or neoplastic transformation.

## Author Contributions

AZ, RM, CF, ASoriani, RP, MC, CC, and ASantoni equally contributed to the manuscript writing.

## Conflict of Interest Statement

The authors declare that the research was conducted in the absence of any commercial or financial relationships that could be construed as a potential conflict of interest.
